# Insulin allergy and resistance successfully treated by desensitisation with Aspart insulin

**DOI:** 10.1186/1476-7961-3-16

**Published:** 2005-12-23

**Authors:** Victor Matheu, Eva Perez, Marta Hernández, Elisa Díaz, Ricardo Darias, Abel González, Jose C García, Inmaculada Sánchez, Laura Feliciano, Agueda Caballero, Fernando de la Torre

**Affiliations:** 1 Medical Inflammation Research, Lund University, Sweden; 2 Allergy, Hospital Universitario NS Candelaria, Spain; 3 Endocrinology Service, Hospital Universitario de Canarias, Spain; 4 Endocrinology, Hospital Universitario NS Candelaria, Spain; 5 Dermatology Service, Hospital Universitario NS Candelaria, Spain

## Abstract

A 25-year-old, with type I Diabetes Mellitus with a previous diagnosis of Protamine Allergy but not to human Insulin, started to notice anaphylactic reactions inmmediatly after bolus with Insulin. Skin prick and intradermal test were positive to all insulins. Skin tests to other potential allergens resulted negative. Examination after bolus of Human Insulin revealed urticaria. Daily insulin requirement were around 2-2,4 U/Kg/day. Slow desensitisation with Aspart insulin, the insulin with lowest size of skin test, was performed using subcutaneous insulin pump. Six months after the end of desensitisation his daily insulin requirement decreased to 0.8 U/Kg/day and oral corticosteroids are being reduced with no symptoms.

## Introduction

Drug hypersensitivity reactions by IgE-mediated mechanisms during therapies have been reported elsewhere. Diagnosis is based in a meticulous clinical history, but many times needs to be supported by skin and/or serological tests and, even sometimes, drug provocation tests since drug preparations may contain multiple potential allergens that can trigger reactions, including drug itself, carrier proteins [1] or additives [2]. Among drugs triggering allergy reactions, insulin allergy might be one of the hot and controversial topics, since it is usually the only known treatment in the case of patients suffering type I diabetes. Although the prevalence of suspected insulin allergy have been reported as high as 2.5% [3], diagnosis should be more accurate since less than one third of patients are finally diagnosed of true insulin allergy [4].

## Case report

The patient is a 25-year-old builder with diabetes mellitus who was diagnosed at 23 years of age. Treatment with three daily injections of semi-synthetic human insulin was initially tolerated (Neutral protamine Hagedorn -NPH, Eli Lilly, Indianapolis, IN-: in the morning and in the evening and Lispro -Eli Lilly, Indianapolis, IN- in lunch-time). Glycosilated hemoglobin (HbA_1c_) controls were around 10%. He gradually started to develop wheals and flare local reactions following his injections of insulin. He was referred to Allergy Service of University Hospital NS Candelaria in April 2003. An initial exam at the Outpatient Clinic revealed strong sensitisation to Nickel Sulphate by patch test after 24, 48 and 96 hours. Separately, skin prick test (SPT) and intradermal test (ID) revealed sensitisation to Protamine (ID at 1:1000 dilution) but not to human insulin. Switch to Insulin without Protamine (Lispro -Eli Lilly, Indianapolis, IN) and subcutaneous insulin pump were both recommended. He did not observed any reaction for a 3-month period using Human regular insulin. Next, he started to notice local symptoms that progressively became worst. The symptoms included immediate episodes of wheals and flare reactions far from insertion site of the catheter, with intensive pruritus on palms and soles, and eventually dyspnea. Then, he developed similar symptoms 2–3 hours after bolus and occasionally some subcutaneos nodules.

Late 2004, he was hospitalised because ketoacidosis. Metil-prednisolone (15 mg/day) and hydroxicine were incorporated. Daily insulin requirement increased up to 2,4 U/Kg/day. He was reassessed at the Outpatient Clinic. SPT were positive to all insulins except Aspart (Novo Nordisk, Denmark). Serial ID showed positive test with Aspart at 1/100 dilution, Glargina (Aventis Pharma, Germany) at 1/100, zinc-crystalline insulin at 1/100, Lispro at 1/1,000 and Human regular insulin at 1/100.000. Skin tests to other potential allergens resulted negative. Specific IgE to protamine (0,43 kU/L), and bovine (2,5 kU/L), pork (3,9 kU/L) and human insulin (2,8 kU/L) were identified (negative: <0.35 kU/L; UniCAP, Pharmacia Diagnostics, Uppsala, Sweden). Controlled reexposure with a bolus of Regular Insulin revealed non-tender swelling with flares (5–6 cm of diameter) far from insertion of catheter without other symptoms. A biopsy specimen of the skin revealed subcutaneous edema with infiltrated cells, including eosinophils.

**Figure 1:**
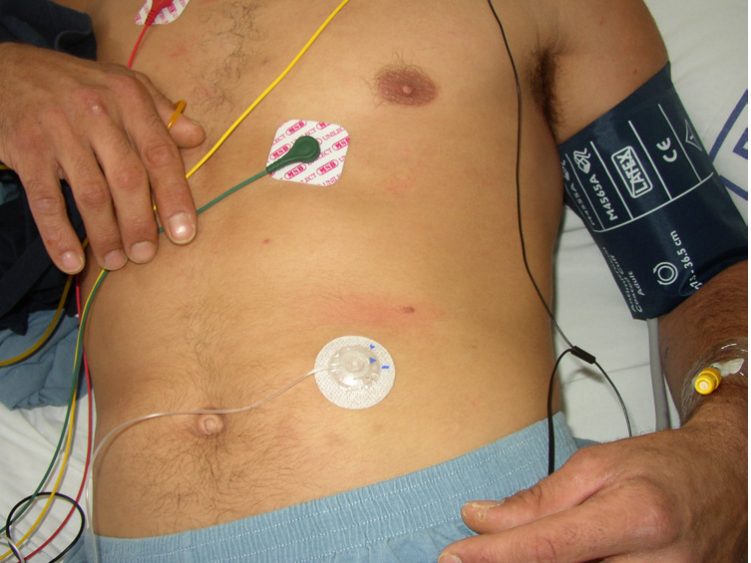
Skin Reaction patient in day #1.

**Figure 2:**
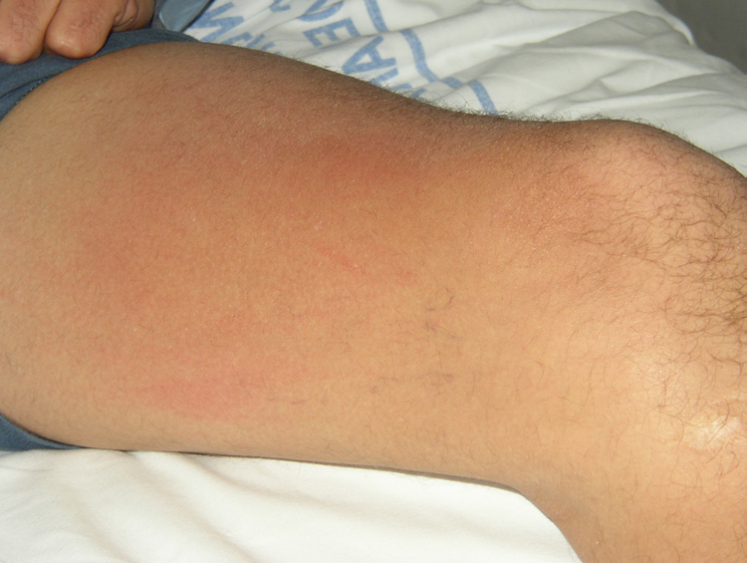
Skin Reaction patient in day #2.

**Figure 3:**
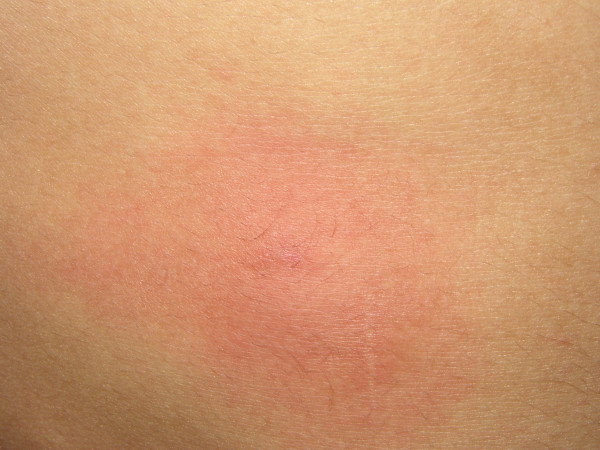
Skin Reaction patient in day #3.

One month later, he was hospitalised because another cetoacydosis episode. As patient had the lowest grade of sensitisation to Aspart Insulin according to skin tests, and rapid insulin was needed, a desensitisation with Aspart insulin was proposed to him. Metil-prednidsolone 30 mg/day, but not hydroxicine, was maintained. Since his high requirement of insulin and risk of ketoacidosis, treatment with endovenous regular insulin was continued up to achieve enough dose of Aspart insulin to sustain him. After written and oral inform consent, desensitisation using subcutaneous insulin pump [5] was performed in 16 sessions (6–7 doses per day). It was started with 0.001 U and increased 2-fold with intervals of 15–30 min, repeating dose in the case of reaction (Table I). During first consecutive 7 sessions patient suffered flare reactions that were cleared up in few minutes without treatment (figure 1, 2 and 3). During all process ketonemia did not show significant changes and serum tryptase levels were normal, although serum histamine did increase 2-fold. Finally enough basal rate was maintained and "square bolus" up to 20 U could be applied without reactions. Six months after the end of desensitisation his daily insulin requirement decreased to 0.8 U/Kg/day and oral corticosteroids are being reduced (metilprednisolone 2 mg/48 h).

**Table 1:** Schedule of desensitization protocol by subcutaneous insulin pump with Aspart Insulin

***DAY***	***DOSE***	***Reaction/n° PULSES***	***TIME BETWEEN DOSES *(min)**
1	0,001	NO/1	15
1	0,005	NO/1	15
1/2	0,01	YES/4	15
1/2	0,02	YES/2	15
2/3	0,05	YES/2	15
2/3/4	0,1	YES/5	15
3/4/5	0,2	YES/6	15
3/4/5/6	0,4	YES/5	15
6/7	0,6	YES/4	15
6/7/8	0,8	YES/4	15
7/8/9	1	YES/3	30
8/9	1,4	YES/4	30
9/10	1,8	NO/4	30
9/10	2,5	NO/4	30
10/11	3	NO/4	30
11/12	3,5	NO/4	30
12/13	4	NO/4	30
13/14	7	NO/4	30
14/15	10	NO/4	30
15/16	15	NO/4	30
16	20	NO/4	30

## Discussion

Multiples cases of allergy to insulin components have been described and reported [6] since it is the treatment for type I diabetic patients. Currently, the prevalence of reactions during insulin treatment seems to be around 2% [3], but less than one third of reported events have been considered related to insulin therapy [4]. Other multiples causes include protamine [7-9], latex[10], cresol, zinc, etc. IgE-mediated reactions have been reported with animal [11] and human insulin [12-15] including semisynthetic and biosynthetic insulins [5,16-21].

**Figure 4:**
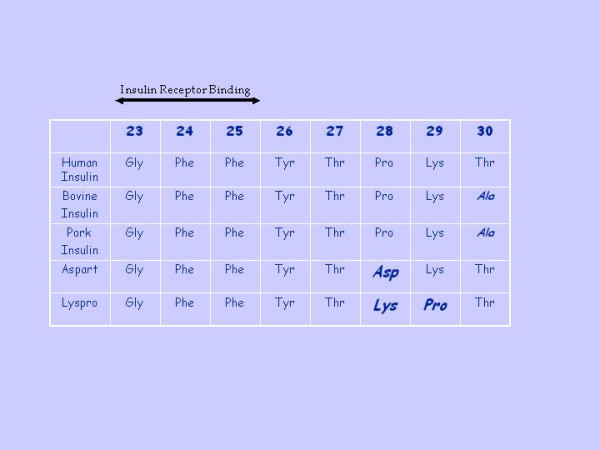
Terminal side of β chain (from aminoacid 23 to 30), including Insulin Receptor Binding (from aminoacid 23 to 25) of different insulins.

Many algorithms of insulin allergy diagnosis have been published although diagnosis is merely based in compatible clinical history and skin test [4] since many diabetic patients can have positive skin test and serum antibodies without clinical symptoms. Switch to insulin analogs has markedly decreased the number of allergic episodes [22-27], since allergenicity of insulin have been proposed by chemical changes in the terminal of B chain [18], where insulin analogs have the modified structure. For instance, Lispro is insulin with lysine and proline in positions 28 and 29, respectively, of beta chain, instead of proline and lysine as in human insulin. Aspart has aspartate in position 28 of beta chain instead of proline (figure 4). Glargine is insulin with arginine and proline in positions 28 and 29 respectively. However, these analogs have also ability to induce allergic episodes[5,16-21,28] and reduced immunogenicity has been more associated to its faster absorption than any changes in the immunogenic epitopes[25].

In this case, insulin pump [5,29] and slow desensitization protocol[30] have demonstrated usefulness to tolerate insulinen and has also helped to reduce insulin requirement as other authors have seen [31]. Although using insulin pump alone have been associated to decrease in insulin dosage [5], our patient was previously using insulin pump with high doses of required insulin. Thus, we could speculate that desensitisation have created a decrease of insulin requirement.

In the near future, anti-immunoglobulin E treatment with omalizumab will probably give another alternative to these patients before transplantation [32]

## Competing interests

The author(s) declare that they have no competing interests.

## Authors' contributions

VM designed Allergy study, was responsible for desensitisation protocol and drafted the manuscript, EP & JC-G performed all skin tests and made the final diagnosis of Insulin allergy, MH has been responsible for patient follow up from the beginning and remitted him to our Allergy Unit, ED & IS performed desensitisation, RD & AG controlled patient's diabetes during hospital stay, LF made skin biopsy, AC controlled patient in Intensive Care Unit every day after desensitisation session and was responsible for coordination trough Medical Transport Ambulance Service between hospitals, FT made first diagnosis of niquel and protamine allergy.
